# Gravity-Driven Membrane Reactor for Decentralized Wastewater Treatment: Effect of Reactor Configuration and Cleaning Protocol

**DOI:** 10.3390/membranes11060388

**Published:** 2021-05-25

**Authors:** Ihtisham Ul Haq Shami, Bing Wu

**Affiliations:** Faculty of Civil and Environmental Engineering, University of Iceland, Hjardarhagi 2-6, IS-107 Reykjavik, Iceland; iuh1@hi.is

**Keywords:** biocarriers, gravity-driven membrane filtration, membrane fouling, periodical membrane cleaning, permeate quality

## Abstract

In this study, three gravity-driven membrane (GDM) reactors with flat sheet membrane modules and various biocarriers (synthetic fibers, lava stones, and sands) were operated for municipal wastewater treatment. The effects of water head, periodically cleaning protocol, and operation temperature on the GDM reactor performance were illustrated in terms of membrane performance and water quality. The results indicated that: (1) the cake layer fouling was predominant (>~85%), regardless of reactor configuration and operation conditions; (2) under lower water head, variable water head benefited in achieving higher permeate fluxes due to effective relaxation of the compacted cake layers; (3) the short-term chemical cleaning (30–60 min per 3–4 days) improved membrane performance, especially when additional physical shear force was implemented; (4) the lower temperature had negligible effect on the GDM reactors packed with Icelandic lava stones and sands. Furthermore, the wastewater treatment costs of the three GDM reactors were estimated, ranging between 0.31 and 0.37 EUR/m^3^, which was greatly lower than that of conventional membrane bioreactors under lower population scenarios. This sheds light on the technical and economic feasibility of biocarrier-facilitated GDM systems for decentralized wastewater treatment in Iceland.

## 1. Introduction

Globally, the annual wastewater production is estimated to be at 359.4 × 10^9^ m^3^, of which ~48% of the total produced wastewater is released into the environment without any treatment [1]. The proportion of untreated wastewater is related to income level, for example, ~72% and ~92% of produced wastewater does not experience any treatment in the lower-middle- and low-income countries, respectively [1]. To mitigate environmental pollution and improve clean water supply, the United Nations (UN) has addressed “access to safe drinking water and sanitation” as one of the UN’s 2030 Sustainable Development Goals (i.e., SDG6) [2]. 

Generally, the conventional activated sludge process (CAS) and membrane bioreactor (MBR) have been widely applied as efficient wastewater treatment processes. However, both treatment processes require high utility, maintenance, and operation costs, which are considered to be major challenges for their application in developing countries or in decentralized regions. In addition, both treatment processes are based on biodegradation of organics/nutrients in wastewater, which generally display lower treatment efficiency at lower temperatures. Thus, CAS and MBR are not favorable wastewater treatment processes in cold climate regions [3,4].

In recent years, gravity-driven membrane (GDM) filtration of municipal wastewater has received great attention as an alternative wastewater treatment process [5,6,7,8,9]. In the GDM systems, the water head beyond the membrane module (i.e., hydrostatic pressure) provides driving force to move water through microfiltration (MF) or ultrafiltration (UF) membranes, without requiring permeation pump [10]. Due to the naturally present biodegradation/biocarrier-facilitated biodegradation as well as membrane separation, the GDM systems can produce treated water with superior quality [7]. Since the GDM systems require low energy consumption and experience less cleaning, they are ideal for the countries/regions where centralized biological wastewater treatment processes are not accessible or favorable [10].

In GDM systems, stabilization of flux can be maintained over a long operational period of time [11]. This phenomenon is associated with eukaryotes’ movement and their predation of prokaryotes, contributing to the heterogeneous biofilm formation on the membrane surface [10,11]. It has been reported that the GDM systems in treating municipal wastewater can achieve the stabilized flux at <5 L/m^2^ h (at water head of 0.3–0.5 m) [5,6,7,9,12], relatively lower than those for treatment of surface water and diluted wastewater [13] and seawater pretreatment [14]. Due to relatively higher organic content in wastewater, organic fouling and proliferation of prokaryotes on the membrane may contribute more greatly to membrane fouling compared to the positive contribution of eukaryotes, leading to lower permeate flux [7].

As the reported GDM systems had different operation conditions and reactor configurations for wastewater treatment, dissimilar membrane fouling mechanisms were observed. In some studies, the cake layer fouling resistance was identified as the predominant contribution to lowering the membrane permeability [5,7,8,12], while other studies highlighted the importance of physically irreversible fouling in determining permeability [6]. In terms of different fouling mechanisms, several strategies have been attempted to improve the permeate flux of GDM systems. For example, the biocarriers [6,7], or coagulation [12], or coagulation with ozonation [8] were integrated with the GDM systems, aiming to pretreat the wastewater before it permeates the membrane. Alternatively, employing membrane relaxation and/or air scouring was performed in the GDM systems for flux enhancement [5]. Nevertheless, the permeate flux levels in GDM systems were still lower than those in conventional MBRs (>10 L/m^2^ h). It has been well known that periodical chemical cleaning is a commonly used strategy in MBRs for maintaining their sustainable operation, but this has not been attempted in GDM systems. In particular, the economic feasibility of GDM systems with periodical chemical cleaning has not been well illustrated. In addition, the influence of variable water head (simulating batch feeding mode under decentralized wastewater treatment scenario) and temperature variation on the GDM performance has been little investigated.

In this study, three GDM systems packed with different biocarriers (synthetic fibers, lava stones, and sands) were employed to treat primary wastewater. The effects of variable water head, periodical chemical cleaning, and operation temperature on the GDM performances were examined in terms of reactor performance, membrane performance, and water quality. The economic analysis of the three GDM systems was performed considering the population size of the decentralized community, with a conventional MBR system as baseline for comparison. 

## 2. Materials and Methods

### 2.1. Experimental Setup and Operation Conditions

**Figure 1:**
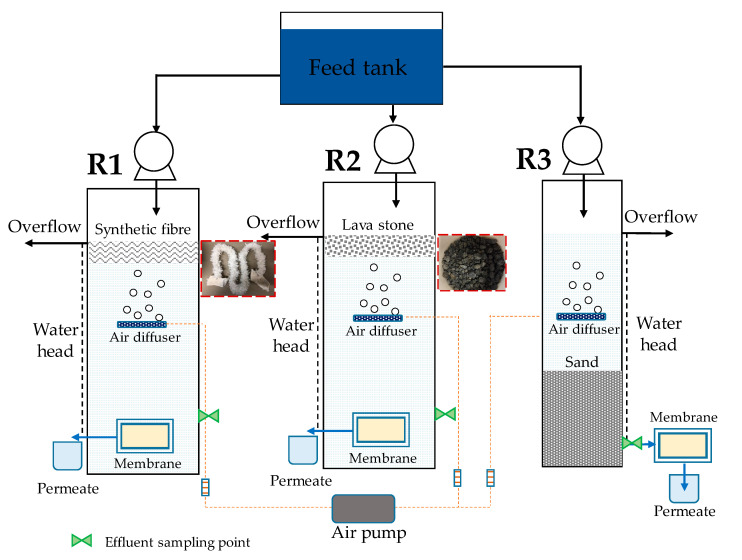
Schematic diagram of the GDM reactors.

As shown in Figure 1, three lab-scale GDM reactors with microfiltration membrane modules (0.2 µm, Microdyn Nadir, Wiesbaden, Germany) were operated in parallel. In detail, reactor 1 and 2 (hereinafter defined as R1 and R2) had the same effective volume of ~7 L. In R1 and R2, synthetic fibers (~0.93 m) and lava stones (~1 kg) were used as biocarriers, respectively, which were packed in a net bag and located on the top of the reactor. The membrane module (membrane area of 36 cm^2^) was submerged into the reactor below the overflow line at 0.25 m. In reactor 3 (hereinafter defined as R3; ~1.4 L), the sand (~1 kg) was packed in the reactor. The membrane module (membrane area of 22 cm^2^) was placed outside of the reactor and the distance between the permeate outlet and overflow line was 0.34 m. In each reactor, the feed water was delivered from a feed tank into the reactor via a peristatic pump (Longer, Baoding, China) and air (0.6 L/min for R1 and R2; 0.2 L/min for R3) was introduced by an air pump into the reactor via an air diffuser, which was placed below the overflow line at ~16 cm. 

The primary treated wastewater was collected from a wastewater treatment plant (Veitur’s plant in Klettagarðar, Reykjavik) and used as the feed water. The GDM reactors were continuously operated for 80 days (divided into four stages, i.e., ~ 20 days per stage), as described in Table 1. 

In stage I, the constant water head (i.e., the distance between the overflow line and membrane permeate outlet) was maintained in each reactor by regulating the feed pump flow rate. The membrane filtration was performed without cleaning at room temperature (~22 °C). In stage II, III, and IV, the feed water was topped up daily to simulate variable water head situation. In stage II, the membrane filtration was performed without cleaning at room temperature (~22 °C). In stages III and IV, periodical chemical cleaning (30 min and 60 min per 3–4 days, respectively) with sodium hypochlorite (NaClO; 0.5%, 50 °C) was performed. For R1 and R2, the cleaning solution (~20 mL, i.e., the volume of the permeate channel) was pumped into the permeate side and permeate flow valve was closed for soaking membrane; for R3, the membrane module was taken from the reactor, and a pump (Longer, Baoding, China) was used to recirculate the chemical solution along the membrane surface at a crossflow velocity of 0.1 m/s to perform cleaning. In stage IV, the low temperature in the reactor (average at ~10 °C) was achieved by periodically placing ice water in the reactor jacket, which was made of insulation materials.

### 2.2. Membrane Flux Measurement and Resistance Analysis

Membrane permeate was collected within a certain filtration time and weighed using a balance (Ohaus, Switzerland). The permeate flux (L/m^2^h, LMH) was calculated by dividing the permeate flow rate (i.e., permeate weight/filtration time) per membrane area. To make a fair comparison, the measured flux J_(T,H)_ at a temperature at T °C and a water height of H (m) was normalized to the flux (i.e., normalized flux) at the temperature at 22 °C and height of 0.25 m (J_(22 °C, 0.25 m)_) using Equation (1):

(1) $$J_{\left(22\;{}^{\circ}C,\;0.25\;m\right)}=\frac{J_{\left(T,H\right)}\times \mu_{T}\times 0.25}{\mu_{22{}^{\circ}C}\times H}$$

where μ_22 °C_ and μ_T_ represent the water viscosity at 22 °C and at the operational temperature T, respectively. The viscosity μ_T_ was calculated according to an empirical equation, Equation (2) [15],

(2) $$\mu_{T}=1.784-(0.05\cdot T)+(0.0011\cdot T^{2})-(10^{-5}\cdot T^{3})$$

The normalized stable flux was obtained by averaging the normalized flux during the last 2 days in each stage. The permeability (P) at the sampling time (t) was calculated as the measured flux J_(T,H)_ divided by the water head of H (m). The averaged permeability was calculated based on Equation (3), in which Δt presents the time duration.

(3) $$\mathrm{Averaged}\;\mathrm{permeability}=\frac{\sum P_{t}\Delta t}{\sum \Delta t}$$

The fouling resistance was examined using a resistance-in-series model based on Darcy’s Law, which was described in our previous studies [16,17]. In detail, the clean membrane (R_m_), cake fouling (R_c_), irreversible fouling (R_ir_), irremovable fouling resistance (R_im_) are calculated as Equations (4)–(7), respectively, in which TMP is water head and μ_T_ is the viscosity of permeate water at temperature T °C.

R_m_ = TMP/(μ_T_ J_m_) (4)

R_c_ = TMP/(μ_T_ J_f_) − TMP/(μ_T_ J_p_) (5)

R_ir_ = TMP/(μ_T_ J_p_) − TMP/(μ_T_ J_c_) (6)

R_im_ = TMP/(μ_T_ J_c_) − TMP/(μ_T_ J_m_) (7)

For each stage, clean membranes were used to filtrate clean water for 30 min before filtrating wastewater, and the clean water flux was measured as J_m_. The wastewater filtration was performed for 20 days and the final permeate flux was defined as J_f_. After that, the cake layer foulants were removed from the membrane surface by a wet cotton stick (i.e., physically cleaning) and then suspended in a tube with 10 mL of clean water (i.e., cake layer foulant sample; note: water performed lubricant to avoid membrane damage. After the yellowish cake layer was removed, the white-colored membrane was clearly observed, showing the removal of cake layer was complete). The physically cleaned membrane was used to filtrate clean water for 30 min and the clean water flux was measured (defined as J_p_). Chemical cleaning was then performed by soaking the membrane in 0.5% of NaClO for 2 h and the clean water flux was measured and defined as J_c_.

### 2.3. Analytical Methods

The pH, conductivity, and dissolved oxygen levels in the feed tank, reactor, and permeate tank were measured using pH, conductivity, and DO meter, respectively (Hach, Loveland, CO, USA). The feed, effluent (i.e., the water fed to the membrane module), and permeate samples were collected periodically and were kept at 4 °C before analysis. The turbidity was analyzed by a turbidity meter (VWR, Radnor, PA, USA). The BOD_5_ was examined based on the standard method [18]. The COD and TN measurements were performed using COD and TN kit (Hach, Loveland, CO, USA), respectively, and were examined by a spectrometer (Hach, Loveland, CO, USA). The total suspended solids (TSS) were analyzed using a spectrometer (Hach, Loveland, CO, USA). A two-sample *t*-test was performed to illustrate the statistical significance and the *p*-value for the two-sample *t*-test at a significance level of 0.05 was presented.

### 2.4. Cost Estimation

To determine the economic feasibility of the proposed GDM systems under the Icelandic scenario, the capital and operational costs were estimated based on the details in Table 2, with a population size of 10–5000 people and local daily personal wastewater production of 270 L/p/d [19]. The reactor hydraulic retention time (HRT) was assumed to be 8 h and the lifespan of GDM systems was assumed to be 20 years. 

## 3. Results and Discussion

### 3.1. Reactor Performance

In this study, the three GDM reactors were continuously operated for 80 days. The water quality in the feed, reactor, and permeate is described in Table 3. In the presence of aeration, the DO levels in the aeration zones of three reactors were maintained at ~7.6–8.2 mg/L, i.e., aerobic conditions. The pH levels in the reactor and permeate were slightly higher than those of the feed (*p* < 0.05), regardless of reactor configuration. The conductivity levels in the feed, reactor, and permeate samples were comparable (*p* > 0.05). Apparently, the turbidity level in the effluent was much lower than that in the feed, showing the averaged removal ratio of ~59%, ~49%, and ~71% for R1, R2, and R3, respectively. Similarly, the effluents in R1, R2, and R3 showed ~66%, ~64%, and 85% of TSS removal from the feed, respectively. As the aeration diffuser was located in the middle of the reactor, the particulates could settle down in the reactor when they left the aeration zone. In particular, in the R3, the sand layer could perform an additional size-exclusion role, resulting in producing an effluent with fewer particulates. This phenomenon has been well illustrated in the previous studies [26,27]. After the effluent passed through the MF membrane, the permeate was almost particulate-free (turbidity at ~0.1 NTU and TSS was not detectable).

For the three reactors, the organic levels (BOD_5_ or COD) in the effluent were comparable (*p* > 0.05; Table 3). In R1, R2, and R3, the averaged BOD_5_ removal ratio was ~67.5%, ~63.9%, and ~63.8%, respectively; the averaged COD removal ratio was ~79.5%, ~74.9%, and ~71.6%. Such organic removals in the reactors could be attributed to biodegradation/biosorption by the attached microorganisms in biocarriers/suspended microorganisms from feed water, as well as organic particulate sedimentation. The organic removal ratios in the reactors were comparable to those in the previously reported GDM reactors, regardless of biocarrier types, reactor configurations, and operating conditions [7,27]. 

In addition, the membrane further removed the BOD_5_ at ~26.7% (R1), ~31.8% (R2), and ~32% (R3); and COD at ~2.1% (R1), ~7% (R2), and ~6.2% (R3), on average. This reveals that the biofilm on the membrane tended to utilize the biodegradable organics in the effluent and produce their metabolic products that cannot be further biodegraded. Such non-biodegradable organic substances passed through the membrane and presented in the permeate, which could be detected by COD measurement instead of BOD_5_ measurement. In several previous GDM studies for pretreating seawater and wastewater treatment, it has been elaborated that the biofilm on the membrane surface can retain and utilize biopolymers (hydrolyzed and/or biodegraded) to produce low molecule weight neutrals [6,14]. 

Nevertheless, the biological degradation/settling behavior of organic matters in the reactor contributed majorly to organic removal compared to membrane separation. The organic levels in the three GDM permeates were also not significantly different (*p* > 0.05; Table 3). This implies that the biocarrier types and reactor configuration may not influence the overall organic removals in the GDM systems. Importantly, the treated water quality in the three GDM systems met the EU urban wastewater discharge standards (COD ≤ 125 mg/L; BOD_5_ ≤ 25 mg/L; TSS ≤ 35 mg/L) [28].

Compared to R1 and R2 (~4.5% TN removal from the feed), R3 could produce the effluent with greatly lower TN level (~21.8% TN removal from the feed) (*p* < 0.05; Table 3). Furthermore, the biofilm on the membrane contributed ~17.7% (R1), ~18.5% (R2), and ~14.9% (R3) of TN removal from the feed. Notably, in three reactors, the feed water was introduced to the top aerobic zone. In R1 and R2, the aerobic biofilm developed on the biocarriers would perform nitrification to convert ammonia in the feed water to nitrate, while the settled bioflocs and biofilm on the membrane surface were expected to perform denitrification under anoxic conditions. In the aeration zone of R3, the suspended bioflocs derived from the feed water performed nitrification, and the sand layer and membrane surface facilitated development of anoxic biofilm for denitrification. Thus, the higher TN removal ratio in R3 (total ~36.7%) could be attributed to the enhanced denitrification by the developed biofilm on the sands. In the previous studies on the granular activated carbon (GAC) facilitated-GDM reactors (the feed water was introduced from the bottom anoxic zone packed with GAC), it was found that employing intermittent aeration in the top aerobic zone could achieve ~29.3–37.3% TN removal [7]. Further implementing internal recirculation could enhance the TN removal ratio (~78.6%) via improving denitrification ratio [6]. Thus, in partially aerated GDM reactors, as denitrification was the limiting step for TN removal, it suggests modifying GDM reactor design to facilitate nutrient mitigation in future study. As required by the EU council [28], the minimum TN level in the discharge wastewater to the sensitive areas (i.e., subject to eutrophication) is set at 10–15 mg/L. The GDM reactors produced permeate with TN levels at 16–20 mg/L; therefore, further improving TN removal is crucial for Icelandic wastewater treatment if the treated wastewater is discharged into the river instead of the ocean. 

### 3.2. Membrane Performance

**Figure 2:**
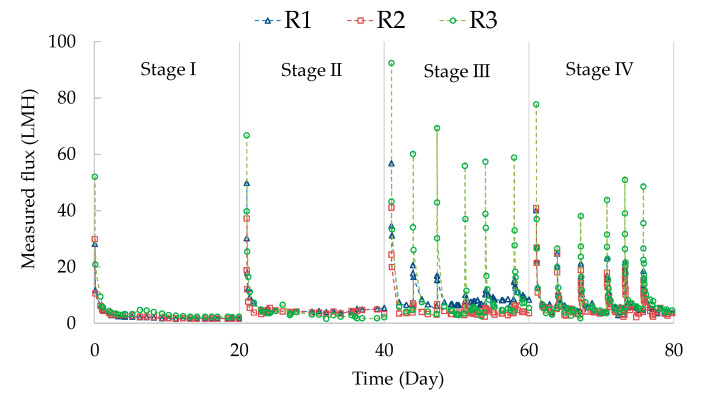
The flux development profiles in the GDM reactors.

**Figure 3:**
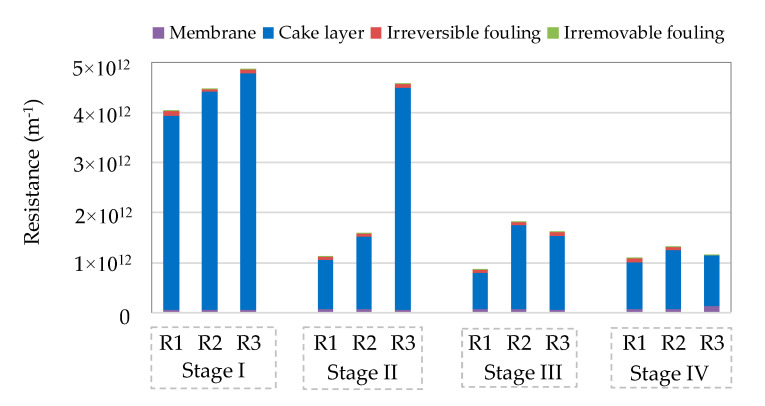
Fouling resistance distribution in the GDM reactor.

The permeate fluxes were recorded through 80-day operation and are presented in Figure 2. During the continuous filtration (Stage I and II), the permeate fluxes experienced a two-stage pattern, i.e., a dramatically decreasing trend during the initial 1–2 days, followed by relatively constant development. In Stage III and IV, periodical chemical cleaning was applied to the membrane, which led to recovery of the permeate flux. After that, the permeate flux also displayed the two-stage pattern between two cleaning cycles. At the end of each stage, the distribution of fouling resistance was examined, as shown in Figure 3. Clearly, the cake layer fouling was the predominant fouling mechanism (>~85%) in the GDM systems, regardless of reactor configuration and operation conditions. A similar observation was also reported in other studies on the GDM systems for municipal wastewater treatment [5,7,8,12]. Researchers [5,7] further pointed out that the cake layer fouling potential was associated with the amount of biopolymers deposited on the membrane surface. It was thought that these biopolymers contain transparent exopolymer particles (gel-like acidic polysaccharides), which may facilitate biofilm development and limit eukaryotic predation/movement behaviors [6,29]. 

**Figure 4:**
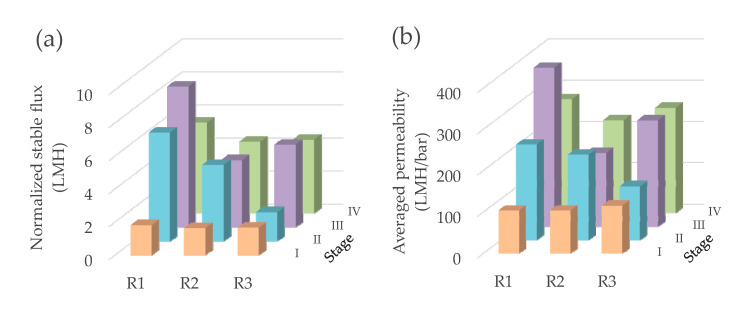
(**a**) The normalized stable flux and (**b**) averaged permeability in the GDM reactors.

To make a fair comparison, the normalized stable flux (J_(22°C, 0.25m)_) and averaged permeability were calculated and are described in Figure 4. In Stage I (i.e., the constant water head), the normalized stable fluxes in the three GDM reactors were relatively comparable, at 1.82, 1.64, and 1.67 LMH for R1, R2, and R3, respectively. With variable water head (Stage II), the normalized fluxes of R1 (6.59 LMH) and R2 (4.63 LMH) were higher than that of R3 (1.77 LMH). In previous GDM studies [14,30], it has been illustrated that the higher water head (i.e., higher hydrostatic pressure) would lead to more cake layer resistance. As illustrated by Fortunato et al. [5], the biofilm cake layer developed on the membrane surface of the GDM system displayed an elastic structure. Thus, a higher driving force would possibly facilitate compaction of such a cake layer. By varying water head, it was expected to perform as “filtration-relaxation” for the cake layers. It is noted that the water head in R3 (0.24–0.34 m) was higher than those in R1 and R2 (0.19–0.25 m). Possibly, in R3, the formed cake layer under a higher water head had a dense nature, which may not be readily relaxed and further self-dispatched from the membrane surface when the water head was lower. As a result, the continuous accumulation of such compacted cake layers on the membrane could cause a lower permeate flux in R3.

**Figure 5:**
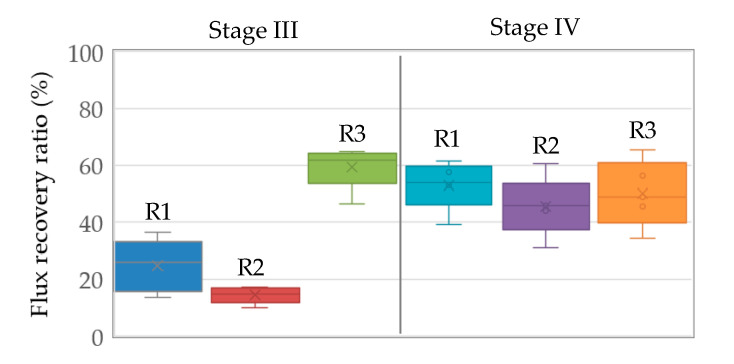
The permeate flux recovery by periodical chemical cleaning.

In the GDM systems, it is noted that the cake layers formed on the membrane surface were associated with microbial community development and biopolymer accumulation [7]. On the other hand, the submerged flat sheet organic membrane modules were limited by pressurized physical backwashing. Thus, periodical chemical cleaning (30–60 min per 3–4 days) was employed for the GDM reactors, aiming to inhibit the development of biofouling on the membrane (in R1 and R2, the chemical solution in the permeate side for soaking membrane; in R3, chemical solution flushing the membrane surface). Clearly, periodical chemical cleaning would benefit permeate flux recovery (Figure 2) and the recovery ratios are associated with cleaning modes and duration (Figure 5). 

In Stage III (30 min of cleaning duration), the permeate flux recovery ratios in R1, R2, and R3 were averaged at ~24.8%, ~14.4%, and ~59.5%, respectively. It was thought that besides the chemical role, physical flushing applied in R3 could provide additional shear force to remove cake layer, achieving more effective permeate flux recovery. Furthermore, the chemical cleaning duration was extended to 60 min in Stage IV. The permeate flux recovery ratio was improved to ~53.1% and ~45.6% in R1 and R2, respectively. Previous research work [31,32] has illustrated that temperature plays a crucial role in determining cake layer morphology and production of extracellular polymer substances (EPS). In this study, the fouling resistance slightly increased (R1) or decreased (R2) with decreasing operation temperature from Stage III to IV (Figure 3). Thus, the improved recovery ratio may not be attributed to the morphology of cake layer/EPS production formed under various temperatures; indeed, possibly the extended soaking time allowed the chemical solution more efficient diffusion through membrane from the permeate side to the cake layer. However, in R3, extending cleaning duration from 30 min to 60 min did not lead to a further increase of the permeate flux recovery ratio. Possibly, the residual cake layer could be tightly attached on the membrane, which could not be flushed from the membrane surface even extending flushing duration.

Although the permeate flux was recovered after a short-term chemical cleaning, it still dropped significantly within one day and then appeared to be stable (Figure 2). A comparison of the normalized stable fluxes in Stage III and IV (Figure 4) showed that the normalized flux followed a sequence of R1 > R3 > R2. This revealed that the normalized stable flux was independent from the recovery ratios (Figure 5). In addition, the permeate flux in R1 decreased when the operation temperature dropped from ~22 °C (Stage III) to ~10 °C (Stage IV), while R2 and R3 achieved relatively comparable fluxes under both temperature conditions. It is recalled that the organic levels of the effluents were comparable in the three GDM reactors (Table 3). The dissimilar effects of temperature on permeate flux may be associated with the organic composition of the effluent, which was related to the microbial community developed on the different biocarriers. As the employed biocarriers were not sterilized, the Icelandic lava stones and sands may have immobilized certain psychrophiles (note: in Iceland, the mean annual temperature is at ∼6 °C) [33], which could perform their activity under lower temperature conditions. Further investigation is needed in the future.

As shown in Figure 4b, the averaged water permeability in this study ranged from ~104 to 231 LMH/bar without periodical chemical cleaning (Stage I and II) and at ~178–385 LMH/bar with periodical chemical cleaning (Stage III and IV). The water permeability values during Stage II–IV (variable water head alone, or variable water head with periodical chemical cleaning) were higher than those in the previously reported GDM systems for municipal wastewater treatment (~44–150 LMH/bar under constant water head without periodical chemical cleaning) [6,7,9,11,12]. This highlights that employing variable water head could benefit improvement of permeate flux. Generally, the decentralized wastewater treatment facility (especially community-level facility) receives variable wastewater flow, which allows operation of GDM systems under variable water head. 

### 3.3. Cost Estimation

**Figure 6:**
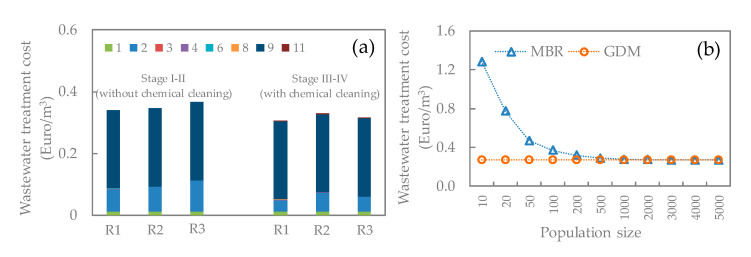
(**a**) Water production cost of GDM systems. The numbers shown in the legend represent the cost items shown in Table 2. Capital cost item: 1—reactor tank; 2—membrane; 3—feed pump; 4—air blower (for biological); 6—biocarriers. Operational cost item: 8—feed pump energy cost; 9—air blower (for biological) energy cost; 11—chemical cost. (**b**) Comparison of water production cost in GDM and MBR systems.

In this study, two scenarios, i.e., without and with periodical chemical cleaning, were considered for estimating the water production cost in three GDM reactors. The averaged permeability in Stage I and II, and that in Stage III and IV was employed to calculate the water production costs under these two scenarios, respectively. Figure 6a shows the cost distribution in the GDM systems. Overall, the water production cost of GDM systems ranged from 0.31 to 0.37 EUR/m^3^, in which aeration energy cost (~70–83%) and membrane cost (~12–28%) were major cost items. To further reduce wastewater treatment cost, decreasing air flow rate in the GDM reactor was proposed, which needs to be further examined in terms of reactor performance and water quality. 

Although employing periodical chemical cleaning requires chemical cost, it benefited in achieving higher permeability (i.e., higher water treatment capacity) (Figure 4b), which in turn led to lower membrane cost. Thus, the GDM systems with periodical chemical cleaning displayed slightly lower water production cost. In addition, the GDM reactor with synthetic fiber as biocarriers (R1) had the lowest water production cost under both scenarios compared to those packed with lava stone (R2) and sands (R3). This was associated with the higher permeability in R1 (Figure 4b), which benefited in reducing membrane cost. However, environmental impacts of chemical discharge and used synthetic fibers need to be further evaluated.

In order to examine the economic feasibility of the GDM systems for decentralized municipal wastewater treatment, a comparison of water production costs of GDM and conventional aerobic MBR systems was performed in terms of population size, as shown in Figure 6b. Clearly, for MBR systems, the water production cost significantly dropped with population size (<500 population) and appeared to be constant under >500 population condition. The higher water production cost at a lower population size was derived from the higher contribution of the capital cost of the control system, which is a necessary component for achieving continuous operation of an MBR system. Whereas the GDM systems are free from the control systems due to their simple operation philosophy. Thus, in the GDM systems, there was almost no influence of population size on the water production cost. Additionally, it is noted that MBR operation requires more experienced manpower due to its complicated nature (such as control system and sludge disposal). Therefore, the GDM systems showed advantages in terms of cost and easy operation nature. 

## 4. Conclusions

This study illustrated the GDM reactor performance under different reactor configurations (synthetic fibers, lava stones, and sands as biocarriers) and operation conditions (constant water head vs. variable water head), periodical cleaning protocol, and operation temperature. The experimental results revealed that the variable water head and periodical short-term chemical cleaning could increase water productivity. The type of biocarriers had negligible effect on the permeate water quality based on organic and nutrient levels. Whereas the biocarriers located in the anoxic zone could enhance nitrogen removal due to possibly improved denitrification. When Icelandic lava stones and sands were employed as biocarriers in the GDM reactors, the membrane permeability was not influenced by varying temperature. The cost estimation also highlighted that the wastewater treatment costs of the biocarrier-facilitated GDM reactors were less than 0.37 EUR/m^3^, which are more suitable for decentralized wastewater treatment scenarios. 

## Figures and Tables

**Table 1:** The operation conditions of GDM reactors.

Stage	Operation Parameters	R1	R2	R3
Stage I (day 0–20)	Condition	Operated at ~22 °C and no cleaning		
	Constant water head	0.25 m	0.25 m	0.34 m
Stage II (day 21–40)	Condition	Operated at ~22 °C and no cleaning		
	Variable water head	0.19–0.25 m	0.19–0.25 m	0.23–0.34 m
Stage III (day 41–60)	Condition	Operated at ~22 °C and periodical chemical cleaning (30 min per 3–4 days)		
	Variable water head	0.21–0.25 m	0.21–0.25 m	0.24–0.34 m
Stage IV (day 61–80)	Condition	Operated at ~10 °C and periodical chemical cleaning (60 min per 3–4 days)		
	Variable water head	0.22–0.25 m	0.22–0.25 m	0.24–0.34 m

**Table 2:** The details of capital and operational cost estimation.

Capital Cost Item	Details
(1) Reactor tank (GDM, MBR)	Feed flow*HRT*1.1*220 EUR/m^3^ (1.1 is a factor considering biocarrier volume and submerged membrane volume based on the lab-scale biocarrier packing situation and flat sheet membrane packing density at 360 m^2^/m^3^ [20]; tank price was assumed to be 220 EUR/m^3^)
(2) Membrane (GDM, MBR)	((Feed flow)/(permeate flux)) * 50 EUR/m^2^ (For GDM, permeate flux = averaged permeability*water head at 0.25 m; for MBR, permeate flux was set at 20 LMH; MF membrane price was assumed to be 50 EUR/m^2^ [21])
(3) Feed pump (GDM, MBR)/permeate pump (MBR)	Feed flow*20 EUR/m^3^/h [22]
(4) Air blower (for biological) (GDM, MBR)	Feed flow*75 EUR/m^3^/h [22]
(5) Air blower (for membrane in MBR)	Feed flow*80 EUR/m^3^/h [22]
(6) Biocarriers (GDM)	Reactor volume*packing ratio* biocarrier cost (Packing ratio was estimated based on the lab setup and biocarrier cost was estimated at 0.255 EUR/m for synthetic fiber, 20 and 52 EUR/ton for lava stone and sands)
(7) PLC control system (MBR)	(20,000 + feed flow*10) EUR [23]
**Operational Cost Item**	**Details**
(8) Feed pump energy cost (GDM, MBR)/permeate pump (MBR)	((Water head*feed flow*365/(36*pump efficiency))*0.134 EUR/kWh (Pump efficiency is assumed at 80%; for GDM, the feed pump water heat at 25 cm (i.e., 0.023 bar); for MBR permeate pump, pressure (water head) at 0.2 bar; electricity cost in Iceland was at 0.134 EUR/kWh)
(9) Air blower energy cost (for biological) (GDM, MBR)	Aeration rate * 0.025 kWh/m^3^ air * 0.134 EUR/kWh [24]
(10) Air blower energy cost (for membrane in MBR)	Membrane area * 0.019 kWh/m^3^ air * 0.3 m^3^ air/m^2^h *0.134 EUR/kWh [24,25]
(11) Chemical cost (GDM, MBR)	0.024 L/m^2^/time* membrane area* 60 EUR/m^3^ *100 time/year
(12) Sludge treatment (MBR)	Biomass (5 g/L) * reactor volume* 150 EUR/ton dry mass (SRT at 20 days) [21]

**Table 3:** Water quality in the feed, effluent, and permeate.

Parameter	Feed Water	R1		R2		R3	
		Reactor (Effluent)	Permeate	Reactor (Effluent)	Permeate	Reactor (Effluent)	Permeate
DO in aeration zone (mg/L) (n = 27)	-	8.0 ± 1.1 (7.1, 8.9) ^1^	-	8.2 ± 0.9 (7.8, 9.0)	-	7.6 ± 0.6 (6.1, 8.3)	-
pH (n = 28)	7.8 ± 0.4 (6.8, 8.6)	8.2 ± 0.4 (6.9, 8.9)	8.8 ± 0.5 (7.8, 9.4)	8.1 ± 0.4 (6.8, 8.7)	8.7 ± 0.4 (7.8, 9.3)	8.1 ± 0.4 (7, 8.8)	8.6 ± 0.3 (7.7, 9.1)
Conductivity (μS/cm) (n = 28)	1231 ± 373 (955, 1893)	1193 ± 299 (932, 1842)	1196 ± 289 (887, 1830)	1213 ± 311 (938, 1877)	1209 ± 302 (868, 1851)	1231 ± 389 (964, 1941)	1235 ± 357 (992, 1967)
Turbidity (NTU) (n = 28)	35.8 ± 20.3 (2.7, 76.3)	8.3 ± 6.9 (0.6, 20.2)	0.1 ± 0.2 (0, 0.8)	11.1 ± 10 (0.7, 50.5)	0.1 ± 0.1 (0, 0.5)	7.0 ± 5.5 (1.3, 19.3)	0.1 ± 0.2 (0, 0.5)
TSS (mg/L) (n = 4)	17.5 ± 7.2 (11, 26)	5.0 ± 2.9 (2, 8)	ND ^2^	7.8 ± 8.3 (1, 18)	ND	2.5 ± 2.4 (1, 6)	ND
BOD_5_ (mg/L) (n = 10)	67.4 ± 27.3 (10, 109)	22.4 ± 10.7 (10.2, 40.2)	2.9 ± 1.8 (0.3, 6.3)	26.5 ± 9.8 (4, 41)	2.1 ± 1.5 (0.6, 5.5)	27.3 ± 11.7 (4, 41)	2.2 ± 1.3 (0.6, 5.5)
COD (mg/L) (n = 4)	110.5 ± 17.9 (90.7, 134)	22.1 ± 6.3 (13, 26.3)	20.2 ± 4.1 (16.5, 24.5)	27.1 ± 2.9 (24.5, 31.1)	19.7 ± 1.5 (18.3, 21.2)	30.1 ± 6.4 (24, 37.5)	24.6 ± 8.5 (17.6, 36.5)
TN (mg/L) (n = 4)	25.1 ± 1.4 (23.5, 26.8)	24 ± 1 (22.5, 24.9)	19.5 ± 1 (18.2, 20.6)	23.9 ± 0.5 (23.4, 24.4)	19.2 ± 1.2 (17.7, 20.3)	19.6 ± 2.8 (15.7, 22.4)	15.8 ± 2 (14.5, 18.7)

^1^ The data in the bracket indicate min and max values respectively; ^2^ ND: not detectable.

## Data Availability

The data presented in this study are available on request from the corresponding author.
